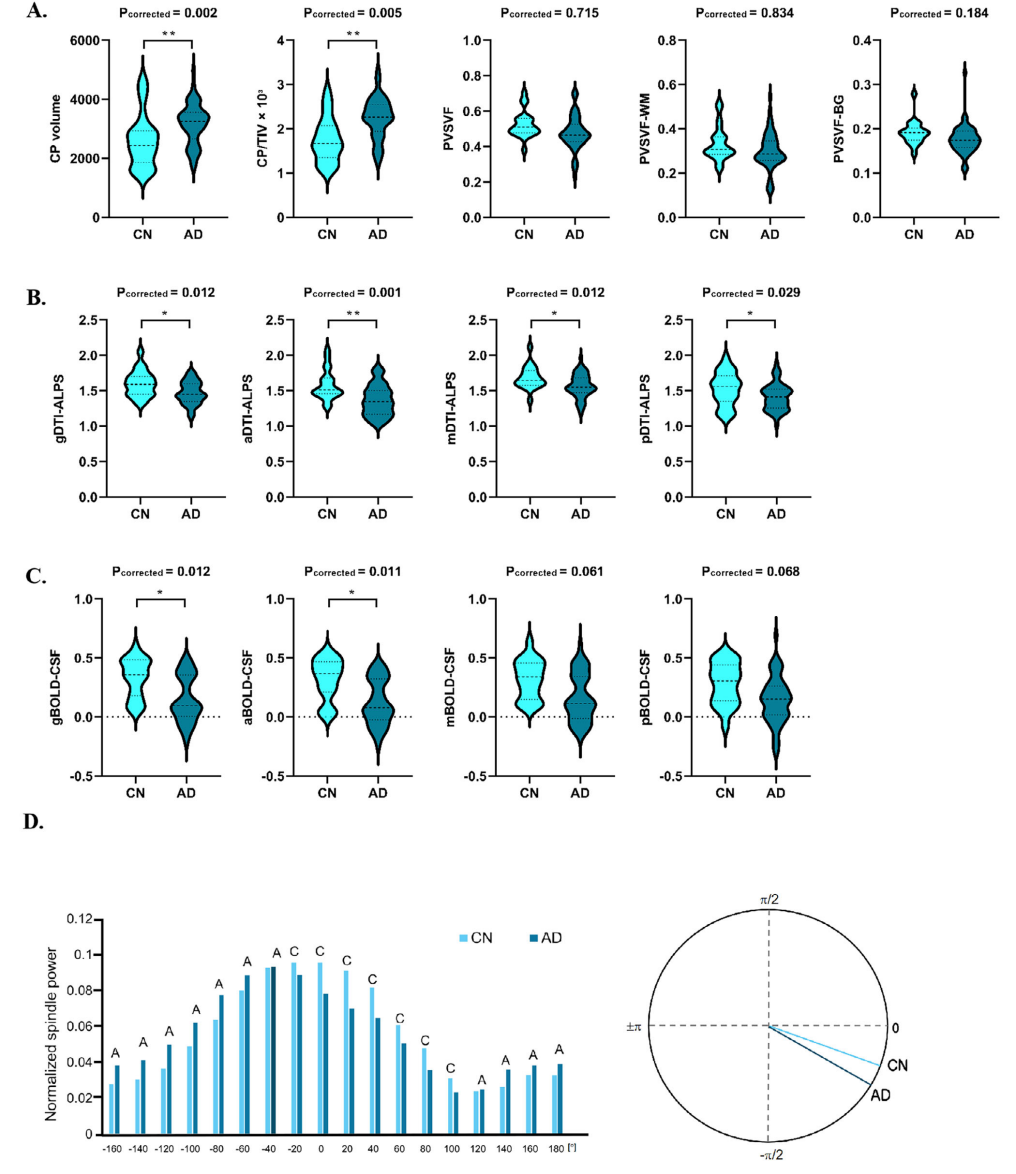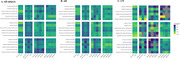# Impairment of glymphatic function supported by sleep oscillatory circuits in Alzheimer's disease

**DOI:** 10.1002/alz70857_100800

**Published:** 2025-12-25

**Authors:** Xiaoduo Liu, Tao Wei, Yi Tang

**Affiliations:** ^1^ Xuanwu Hospital, Capital Medical University, Beijing, Beijing, China; ^2^ Department of Neurology & Innovation Center for Neurological Disorders, Xuanwu Hospital, Capital Medical University, National Center for Neurological Disorders, Beijing, Beijing, China; ^3^ Neurodegenerative Laboratory of Ministry of Education of the People's Republic of China, Beijing, Beijing, China

## Abstract

**Background:**

Comprehending the influence of sleep oscillatory circuits on the glymphatic system is essential for unraveling the neurophysiological mechanisms underlying cognitive decline linked to Alzheimer's disease (AD).

**Method:**

We collected 54 patients with AD and 21 age‐ and sex‐matched cognitively normal (CN) controls. All participants underwent a comprehensive neuropsychological evaluation, overnight polysomnography, multimodal imaging acquisition and biomarker acquisition. Sleep oscillatory events include slow oscillations (SOs), theta bursts, and sleep spindles, as well as their interactions, such as SO–theta burst coupling and SO–spindle coupling. Glymphatic indices including choroidal plexus (CP) volume, perivascular spaces (PVSs), diffusion tensor imaging along the perivascular perimeter (DTI‐ALPS) index, and blood oxygen level‐dependent signal coupled to cerebrospinal fluid signal (BOLD‐CSF coupling) were compared between the two groups.

**Result:**

AD patients exhibit worse glymphatic function and poorer accuracy of SO‐spindle coupling. AD patients had a higher percentage of CP volume (*p* = 0.005), whereas DTI‐ALPS (*p* = 0.012), BOLD‐CSF coupling (*p* = 0.019) and SO phase at peak of spindle (*p* = 0.029) were worse (Figure 1). Correlation analysis showed that global DTI‐ALPS was negatively correlated with SO phase at peak of spindle (*r* = 0.370, *p* = 0.008). Global BOLD‐CSF coupling was positively correlated with the amplitude of theta bursts (*r* = 0.551, *p* = 0.043) in CN population; global DTI‐ALPS showed a positive correlation with the frequency of SO‐spindle coupling (*r* = 0.690, *p* = 0.006). This correlation was attenuated in AD patients (Figure 2). Furthermore, we found that global DTI‐ALPS mediated the association between SO phase at peak of spindle and cognitive decline or reduction in biomarkers. This reflects the role of sleep neural circuit structure in driving the glymphatic system.

**Conclusion:**

Our findings provide crucial evidence for potential neural interactions between sleep oscillatory circuits and the glymphatic system, with this effect being diminished in Alzheimer's disease (AD) patients. Specifically, the reduced precision of SO‐spindle coupling in AD patients may represent a key mechanism driving the impaired glymphatic clearance of white matter, contributing to cognitive decline.